# Shared Genetic Etiology of Autoimmune Diseases in Patients from a Biorepository Linked to De-identified Electronic Health Records

**DOI:** 10.3389/fgene.2016.00185

**Published:** 2016-10-20

**Authors:** Nicole A. Restrepo, Mariusz Butkiewicz, Josephine A. McGrath, Dana C. Crawford

**Affiliations:** ^1^Department of Epidemiology and Biostatistics, Case Western Reserve UniversityCleveland, OH, USA; ^2^Vanderbilt Eye Institute, Vanderbilt University Medical CenterNashville, TN, USA; ^3^Institute for Computational Biology, Case Western Reserve UniversityCleveland, OH, USA

**Keywords:** autoimmune disease, Crohn's disease, rheumatoid arthritis, multiple sclerosis, electronic health records

## Abstract

Autoimmune diseases represent a significant medical burden affecting up to 5–8% of the U.S. population. While genetics is known to play a role, studies of common autoimmune diseases are complicated by phenotype heterogeneity, limited sample sizes, and a single disease approach. Here we performed a targeted genetic association study for cases of multiple sclerosis (MS), rheumatoid arthritis (RA), and Crohn's disease (CD) to assess which common genetic variants contribute individually and pleiotropically to disease risk. Joint modeling and pathway analysis combining the three phenotypes were performed to identify common underlying mechanisms of risk of autoimmune conditions. European American cases of MS, RA, and CD, (*n* = 119, 53, and 129, respectively) and 1924 controls were identified using de-identified electronic health records (EHRs) through a combination of International Classification of Diseases, Ninth Revision, Clinical Modification (ICD-9-CM) billing codes, Current Procedural Terminology (CPT) codes, medication lists, and text matching. As expected, hallmark SNPs in MS, such as *DQA1* rs9271366 (OR = 1.91; *p* = 0.008), replicated in the present study. Both MS and CD were associated with *TIMMDC1* rs2293370 (OR = 0.27, *p* = 0.01; OR = 0.25, *p* = 0.02; respectively). Additionally, *PDE2A* rs3781913 was significantly associated with both CD and RA (OR = 0.46, *p* = 0.02; OR = 0.32, *p* = 0.02; respectively). Joint modeling and pathway analysis identified variants within the KEGG NOD-like receptor signaling pathway and Shigellosis pathway as being correlated with the combined autoimmune phenotype. Our study replicated previously-reported genetic associations for MS and CD in a population derived from de-identified EHRs. We found evidence to support a shared genetic etiology between CD/MS and CD/RA outside of the major histocompatibility complex region and identified KEGG pathways indicative of a bacterial pathogenesis risk for autoimmunity in a joint model. Future work to elucidate this shared etiology will be key in the development of risk models as envisioned in the era of precision medicine.

## Introduction

Autoimmune (AI) and immune-mediated (IM) diseases are a driving force behind disability in the United States with 5–8% of the population affected (2002). Currently there are upwards of 80 conditions that occur as a consequence of immunological attacks on the body's tissues and organs. As a whole, AI and IM diseases share many epidemiological and pathogenic similarities. Most notable is the occurrence of a skewed sex distribution with females more likely to be affected by an AI disease. The female sex ratio of cases is most extreme in Sjögren syndrome (9:1) that occurs as a result of immune-mediated attack on salivary and lacrimal glands in comparison to type 1 diabetes which is sex neutral (Gale and Gillespie, 2001). Over the last decade, genome-wide association studies have consistently identified genes within the major histocompatibility complex (MHC), located on the short arm of chromosome 6, strong regulators of disease risk in a number of AI diseases such as multiple sclerosis (MS) (International Multiple Sclerosis Genetics Consortium et al., 2007, 2011; Patsopoulos et al., 2011), rheumatoid arthritis (RA) (Plenge et al., 2007; Raychaudhuri et al., 2008; Okada et al., 2012), and vitiligo (Jin et al., 2010; Quan et al., 2010). Despite these commonalties, their underlying genetic and molecular profiles vary and are hindered by low case counts with most AI diseases rarely occurring in the general population (i.e., Addison's disease prevalence estimated at ~1 in 20,000). Elusive and overlapping symptoms further confound diagnosis of a condition, often leading patients on a long and costly diagnostic odyssey. Additionally, time to diagnosis, time between presentation of first symptoms to confirmed diagnosis, can approach a year in conditions such as amyotrophic lateral sclerosis (Paganoni et al., 2014).

Large-scale studies for AI and IM diseases typically require recruiting and consenting patients from specialty clinics and individual medical practices, all of which necessitates considerable financial investment and manpower. In recent years, researchers have begun to utilize electronic health records (EHR) for use in clinical and genetic association studies covering a range of conditions from type 2 diabetes (Ng et al., 2014) to response to drugs or treatment (Oetjens et al., 2014; Laper et al., 2016). Use of EHRs rapidly facilitates biomedical studies by providing researchers with access to a repository of extensive, longitudinal medical data. In particular, EHRs offer the opportunity to assess less common conditions such as MS by directly accessing data from specialty disease clinics within the framework of a health care organization. In conjunction with DNA repositories, it becomes feasible to combine genetic and phenotypic data in order to study the immunogenetic architecture of autoimmune disease (Goris and Liston, 2012), much of which is still unknown.

Individuals with one AI disease are at greater risk to develop another AI condition; RA and type 1 diabetes have been observed to occur jointly in patients from a United Kingdom cohort, while there is reduced comorbidity between RA and MS (Somers et al., 2009). Additionally, a GWAS study of inflammatory bowel disease, which includes CD and ulcerative colitis, found variants initially identified in GWAS studies of MS and RA (Liu et al., 2015). In order to delineate potential, common genetic pathways involved in AI and IM disease, we have performed a targeted genetic association study of SNPs known to be associated with AI (MS and RA) and IM (Crohn's disease or CD) in a European American population extracted from the Vanderbilt University Medical Center's (VUMC) DNA biorepository linked to de-identified EHRs. Based on previous evidence from GWAS (Sivakumaran et al., 2011) and candidate gene studies, we hypothesized that MS, RA, and CD share genetic factors within the MHC region, known to include many immune system genes, and outside the MHC region where less is known about genes that may play a role in autoimmunity. Elucidating the role that genetics plays in disease risk and progression is vital for future studies to incorporate risk of a comorbid disorder and lead to better screening and prevention strategies.

## Materials and methods

### Study population

The study population is derived from BioVU, the VUMC biorepository linked to de-identified EHRs. The Synthetic Derivative or SD refers to the de-identified version of Vanderbilt's EHR and contains inpatient and outpatient medical records from VUMC and affiliated clinics. Patient records consist of both structured (e.g., billing codes, procedure codes, laboratory values) and unstructured (e.g., clinical free text) data. The VUMC EHR contains over 2.2 million records with each record containing 1–1000 International Classification of Diseases, Ninth Revision, Clinical Modification (ICD-9-CM) codes captured electronically since the inception of the VUMC EHR more than twenty years ago (Crawford et al., 2015). The SD is linked with VUMC's DNA repository known as BioVU (Roden et al., 2008). These DNA samples are extracted from discarded blood samples collected from outpatient clinical laboratories. Genomic data generated on BioVU samples are available for additional analyses by other investigators after IRB approval. All procedures were approved by the Vanderbilt University's Institutional Review Board that determined that this study met the criteria of “non-human subjects” as no personal identifying information is available to the investigators (The Code of Federal Regulations, 45 CFR 46.102 (f)).

### Phenotype definitions

Detailed protocols for the identification of MS, RA, and CD phenotypes in this EHR have been previously published (Ritchie et al., 2010). Briefly, cases and controls were defined as follows.

#### Multiple sclerosis cases

Cases of MS were extracted from the SD based on the presence of either (1) ICD-9-CM for MS (ICD-9-CM 340) or (2) ICD-9-CM for demyelinating disease of the central nervous system (341.9), transverse myelitis (323.9) AND presence of an MS medication (interferon-beta 1a, interferon-beta 1b, glatiramer, natalizumab, Rituxan) AND a text mention of “multiple sclerosis” AND no mention of an ICD-9-CM for a potentially overlapping autoimmune condition. Due to the complex diagnosis process for MS, all MS cases were manually reviewed. The MS case algorithm deployed here had a positive predictive value (PPV) of 90%.

#### Rheumatoid arthritis cases

RA cases contained all of the following: (a) RA ICD-9-CM (714, 714.0, 714.1, or 714.2), (b) RA medication (i.e., methotrexate, sulfasalazine, minocycline, hydroxychloroquine, adalimumab, etanercept, infliximab, gold, azathioprine, rituximab, anakinra, abatacept, leflunomide), (c) text match for “rheumatoid arthritis,” and (d) does NOT contain an ICD-9-CM for a potentially overlapping autoimmune condition. A randomized, subset of RA cases (*n* = 30) was manually reviewed for quality assurance. The RA case algorithm deployed here had a PPV of 96.6%.

#### Crohn's disease cases

CD cases contained both an ICD-9-CM for CD (555.^*^) and at least one medication for CD (i.e., balsalazide, mesalamine, sulfasalazine, ciprofloxacin, levofloxacin, metronidazole, rifaximin, prednisone, budesonide, azathioprine, mercaptopurine, methotrexate, infliximab, adalimumab, certolizumab, natalizumab). If case records also contained an ICD-9-CM for ulcerative colitis (556.^*^), the ratio of CD ICD-9-CM codes to ulcerative colitis ICD-9-CM codes had to be greater than two to be considered a case in the study. A randomized, subset of CD cases (*n* = 30) was manually reviewed for quality assurance. The CD case algorithm deployed here had a PPV of 100%.

#### Joint controls

Controls were those individuals whose de-identified EHRs met the control criteria for MS, RA, and CD as previously defined (Ritchie et al., 2010). A randomized, subset of 90 controls was manually reviewed for quality assurance. Control records were *devoid* of all of the following:

- Text match for “multiple sclerosis,” no text mention of a conflicting autoimmune disease for CD or RA.
- ICD-9-CM: MS ICD-9-CM, any ICD-9-CM for RA, other inflammatory arthritides, and other conflicting autoimmune conditions, inflammatory bowel disease.
- Medications: MS medications (interferon-beta 1a, interferon-beta 1b, glatiramer, natalizumab, Rituxan).

The PPV of the joint controls was 93.3%.

#### Clinical traits and variables

Demographic data and laboratory tests were extracted and calculated as follows. For cases, age at diagnosis was defined by the date in the patient's EHR for the first mention of an MS (340, 341.9, 323.9), RA (714, 714.0, 714.1, 714.2), or CD (555.^*^) ICD-9-CM code. For controls, age was calculated from date of birth as of 2014. Clinical values for body-mass-index (BMI), rheumatoid factor, and C-reactive protein were calculated as the mean of all measurements in a patient's medical record, regardless of case/control status.

### SNP selection and statistical analysis

Targeted genotype data was pulled from BioVU as of February 2014 for samples that were previously genotyped for other research studies. SNPs selected for analysis were associated with MS, RA, or CD in a published GWAS (at *p* < 10^−6^) identified in the NHGRI GWAS Catalog (Welter et al., 2014) or candidate gene study (at *p* < 0.05) as of 2013 (Supplemental Table 1). In total, genotype data for 345 SNPs in 295 genes or gene regions were accessed and pooled from the following Illumina genotyping arrays: 660W (Denny et al., 2011; Ritchie et al., 2013), 1M (Ng et al., 2014), HumanOmni1-Quad, HumanOmni5-Quad, and Infinium HumanExome BeadChip.

Each SNP considered for analysis was common (minor allele frequency >5%) and passed quality control standards (Hardy Weinberg Equilibrium *p* < 0.001) separately in MS, RA, and CD. Analyses were limited to European American patients, and controls under the age of twenty years were removed from analysis as it cannot be determined if these are true disease-free controls for these later onset conditions. Likewise, cases with age at diagnosis under 20 years were removed from analyses given that the genetic architecture for later onset AI and IM may be different than juvenile onset. Quality control and single SNP tests of association were performed using PLINKv1.07 (Purcell et al., 2007). Each SNP was tested for an association using logistic regression assuming a log-additive genetic model adjusted by age and sex. The Bonferroni significance threshold is set at 1.5 × 10^−4^.

Previous studies have suggested that some autoimmune diseases share a common genetic architecture or associated SNPs have evidence of pleiotropy (Ueda et al., 2003; Criswell et al., 2005; Gough and Simmonds, 2007; Zhernakova et al., 2009; Sivakumaran et al., 2011). To explore whether specific variants are associated with multiple phenotypes in this clinical population, MS, CD, and RA cases were combined into a joint multivariate association test of likelihood ratio for model fit. Ordinal regression adjusted for age and sex was performed on each SNP with MS, RA, and CD modeled as predictors of the genotype. Ordinal regression was performed with the software package MultiPhen (O'Reilly et al., 2012) in R software version 3.2.3 (R Development Core Team, 2013). Twenty-two SNPs from the MultiPhen joint model (i.e., MS, CD, and RA) with a *p* < 0.05 were further characterized using the Pathway Analysis by Randomization incorporating Structure (PARIS), a SNP-based pathway analysis tool that identifies biological pathways significantly enriched by genetic variants adjusted for linkage disequilibrium and gene size (Yaspan et al., 2011).

## Results

### Clinical population characteristics

We extracted 129 CD cases, 119 MS cases, 53 RA cases, and 1642 joint controls from the de-identified EHR. Demographics in Table 1 include information for patients over the age of 20 years for all three phenotypes and joint controls. In general, age and sex distributions were as expected. That is, MS and CD cases were on average younger than controls, while RA cases were on average of a similar age compared with controls. Additionally, MS and RA cases were predominantly female, consistent with the known epidemiology for autoimmune diseases. CD cases were equally likely to be male as female as has been observed in previous epidemiological surveys (Cosnes et al., 2011). Of the MS cases, we were able to manually identify the MS clinical subtype for 48% of patients (Davis et al., 2013; Davis and Haines, 2015). Of these, 42.3% were diagnosed with relapsing-remitting, 33% with secondary progressive, 14% with progressive/relapsing, and 10.2% with primary progressive MS.

**Table 1:** **Demographics of the BioVU multiple sclerosis, rheumatoid arthritis, and Crohn's disease cases and controls by sex**.

	**Multiple sclerosis cases**		**Rheumatoid arthritis cases**		**Crohn's disease cases**		**Controls**	
	**Male**	**Female**	**Male**	**Female**	**Male**	**Female**	**Male**	**Female**
N	33	86	17	36	64	65	805	837
Age in years (SD)	47.6 (15.2)	45.4 (12.8)	60.3 (11.5)	61.3 (12.4)	47.5 (15.2)	49.6 (17.5)	61.1 (15.3)	58.3 (16.6)
BMI, kg/m^2^ (SD)	29.1 (6.2)	27.3 (6.7)	30.3 (4.0)	29.2 (6.7)	26.7 (6.1)	26.8 (7.5)	29.2 (6.7)	29.4 (12.3)
RF, μ/mL (SD)	26.8 (37.1)	16.0 (21.1)	66.5 (60.5)	190.8 (139.3)	7.5 (6.4)	10.5 (7.8)	.	.
CRP, mg/L (SD)	31.4 (50.2)	37.10 (69.3)	12.5 (13.8)	24.9 (53.3)	42.2 (65.1)	20.3 (30.8)	.	.

Values represent means and standard deviations (SD). Abbreviations: RF, rheumatoid factor; CRP, C-reactive protein.

### Validation of EHR phenotype extraction

To further validate the case/control extraction from the EHR, we performed tests of association for each autoimmune disease to replicate previously-reported associations from the literature. We identified 157, 124, and 64 SNPs previously associated with CD, MS, and RA, mostly from GWAS (Supplemental Table 1). Genotype data for these GWAS-identified variants were available for 120 out of 129, 62 out of 119, and 35 out of 53 cases of CD, MS, and RA identified in the EHR, respectively. At a liberal significance threshold of 0.05, we replicated associations for CD (one), MS (11), and RA (one) (Supplemental Tables 2–4).

Despite differences in sample size, previously reported associations proportionally replicated most often in MS (~9%) compared with CD (~1%) and RA (~1.5%) (Table 2). As expected, *HLA-DRA* rs3135388 (De Jager et al., 2009; OR = 3.04; *p* = 0.004) and rs3129889 (Patsopoulos et al., 2011; OR = 3.04; *p* = 0.004), variants in strong linkage disequilibrium with one another, as well as *HLA-DQA1* rs9271366 (Australia New Zealand Multiple Sclerosis Genetics Consortium, 2009; OR = 2.16; *p* = 0.003) were strongly associated with MS in the present study. For CD, we replicated rs7714584 (Franke et al., 2010) located in *IRGM* (OR = 2.66; 95% CI: 1.22-5.78; *p* = 0.013). Additionally, we replicated rs7765379 on chromosome 6 in the RA dataset. SNP rs7765379 was originally identified in a Korean population with an OR of 2.51 (Freudenberg et al., 2011) which is similar to the effect size observed in this study of European Americans (OR = 2.42).

**Table 2:** **Previously-associated SNPs from Crohn's disease, multiple sclerosis, and rheumatoid arthritis that replicated in the present study of European American patients**.

**SNP**	**Gene**	**CHR**	**CA**	**OR (95% CI)**	**CAF_cases_**	**CAF_control_**	**Disease**	***P*-value**
rs7714584	*IRGM*	5	G	2.66 (1.22–5.78)	0.19	0.09	CD	0.013
rs9271366	*DQA1*	6	G	2.16 (1.30–3.62)	0.22	0.14	MS	0.003
rs3135388	*HLA-DRA*	6	T	3.04 (1.40–6.57)	0.25	0.12	MS	0.004
rs3129889	*HLA-DRB1*	6	G	3.04 (1.40–6.57)	0.25	0.12	MS	0.004
rs2243123	*IL12A*	3	C	2.13 (1.16–3.93)	0.36	0.29	MS	0.015
rs2523393	*HLA-B*	6	T	2.77 (2.50–3.08)	0.77	0.56	MS	0.018
rs2546890	*IL12B*	5	A	1.75 (1.32–2.32)	0.63	0.52	MS	0.022
rs12368653	*AGAP2, CYP27B1*	12	A	1.98 (1.10–3.60)	0.57	0.47	MS	0.022
rs4409785	*RGS14*	11	C	1.81 (1.07–3.09)	0.20	0.16	MS	0.027
rs7255066	*PVR*	19	C	1.64 (1.03–2.60)	0.39	0.28	MS	0.035
rs7090512	*IL2RA*	10	C	1.65 (1.01–2.68)	0.34	0.30	MS	0.045
rs7765379	*HLA-DRB1*	6	G	2.40 (1.13–5.13)	0.24	0.11	RA	0.023

Tests of association were performed using logistic regression assuming an additive log model adjusted for age and sex. The threshold for significance was 0.05. For each replicated test of association, the rs number, nearest gene, chromosome (CHR), coded allele (CA), odds ratio (95% confidence interval), coded allele frequency (CAF) for cases and controls, associated disease, and p-value are given.

Coded allele (CA).

Coded allele frequency (CAF).

Odds ratio (OR).

95% confidence intervals (CI).

### Evidence for shared genetic architecture

Known autoimmune disease risk loci, such as the MHC and *PTPN22* (Begovich et al., 2004; Bottini et al., 2004), have been associated with multiple autoimmune diseases such as type 1 diabetes, CD, and MS (Wellcome Trust Case Control Consortium, 2007). Three of the most significant associations identified in this MS study, of the total 332 SNPs tested, were originally associated with CD (Barrett et al., 2008; rs2301436; OR = 1.21) and RA (Kochi et al., 2010; Stahl et al., 2010; rs3093024; OR = 1.19 and rs3093023; OR = 1.13; Table 3). SNPs rs3093024 and rs3093023 in the chemokine C-C motif receptor 6 (*CCR6*) gene, which is located outside of the MHC region yet plays a role in the immune system via B-cell maturation (Bowman et al., 2000), are located approximately 1500 base pairs apart. At least one of these two SNPs has been associated with RA in European-descent (Stahl et al., 2010), Chinese (Jiang et al., 2014), and Japanese (Kochi et al., 2010) populations with similar effect sizes ranging from an OR of 1.13–1.19. In the present study, the two *CCR6* SNPs were associated with MS but in the opposite direction compared with the published RA studies (Table 3).

**Table 3:** **Common genetic variants associated with multiple autoimmune phenotypes**.

**SNP**	**Gene**	**Published phenotype**	**Current study phenotype**	**CHR**	**CA**	**OR (95% CI)**	**CAF_case_**	**CAF_control_**	***P***
rs2301436	*FGFR1OP*	CD	MS	6	A	0.38 (0.22–0.63)	0.38	0.49	0.0002
rs3093024	*CCR6*	RA	MS	6	A	0.39 (0.23–0.66)	0.35	0.47	0.0004
rs3093023	*CCR6*	RA	MS	6	A	0.33 (0.16–0.68)	0.33	0.47	0.002
rs9271366	*HLA-DRB1*	MS and CD	MS	6	G	2.16 (1.29–3.62)	0.22	0.14	0.003
rs2274910	*ITLN1*	CD	MS	1	T	0.36 (0.17–0.77)	0.20	0.34	0.008
rs2836754	*RPSAP64-RPL23AP12*	CD	MS	21	T	1.78 (1.13–2.80)	0.46	0.35	0.011
rs13126505	*BANK1*	CD	MS	4	A	3.09 (1.26–7.6)	0.08	0.07	0.013
rs4409785	*CEP57*	MS and RA	MS	11	C	1.81 (1.07–3.09)	0.20	0.16	0.027
rs6457617	*HLA-DQB1*	RA	MS	6	T	0.60 (0.37–0.96)	0.34	0.49	0.033
rs13031237	*REL*	RA	MS	2	T	1.92 (1.05–3.52)	0.46	0.36	0.034
rs13017599	*NONOP2*	RA	MS	2	A	1.92 (1.04–3.53)	0.46	0.36	0.036
rs2076756	*NOD2*	CD	MS	16	G	0.52 (0.28–0.97)	0.16	0.26	0.041
rs26232	*C5 or f30*	RA	MS	5	T	1.98 (1.03–3.82)	0.39	0.31	0.042
rs9268853	*HLA-DRA*	CD/UC	MS	6	C	0.40 (0.16–0.97)	0.21	0.36	0.043
rs6448432	*LOC105374540*	RA	MS	4	A	0.48 (0.24–0.98)	0.28	0.31	0.045
rs9286879	*LOC105371618*	CD	MS	1	G	0.46 (0.21–0.99)	0.20	0.26	0.047
rs7765379	*HLA-DRB1*	CD and RA	RA	6	G	2.4 (1.12–5.12)	0.24	0.11	0.023
rs10866713	*IL12B*	MS	RA	5	A	4.81 (1.20–19.31)	0.50	0.20	0.026
rs4820425	*RBX1*	CD	RA	22	A	2.10 (1.05–4.21)	0.44	0.26	0.035
rs6556412	*IL12B5*	CD	RA	5	A	2.23 (1.04–4.79)	0.50	0.31	0.038
rs7807268	*RNA5SP249-RPL32P17*	CD	RA	7	G	0.12 (0.01–0.93)	0.10	0.49	0.043
rs12644284	*TRIM2*	MS	RA	4	G	0.35 (0.12–0.97)	0.11	0.27	0.043
rs6952809	*CHST12*	MS	RA	7	T	0.37 (0.14–0.98)	0.15	0.32	0.047
rs11962089	*POPDC3*	MS	RA	6	G	3.10 (1.01–9.51)	0.18	0.08	0.047
rs1323292	*RGS1*	MS	CD	1	C	2.80 (1.48–5.27)	0.36	0.16	0.001
rs2283792	*MAPK1*	MS	CD	22	T	2.58 (1.33–5.01)	0.32	0.46	0.005
rs3780792	*VAV2*	MS	CD	9	G	0.61 (0.41–0.92)	0.26	0.34	0.018
rs2293370	*TIMMDC1*	MS	CD	3	T	0.24 (0.07–0.81)	0.06	0.19	0.021
rs13119723	*IL21*	RA	CD	4	G	0.20 (0.05–0.88)	0.05	0.16	0.033
rs3781913	*PDE2A*	RA	CD	11	A	0.48 (0.25–0.95)	0.26	0.43	0.036
rs7238078	*MALT1*	MS	CD	18	G	0.61 (0.39–0.97)	0.16	0.24	0.036
rs3783637	*GCH1*	RA	CD	14	T	1.58 (1.01–2.47)	0.18	0.12	0.041
rs2002842	*LOC105372221*	RA	CD	18	A	1.45 (1.01–2.08)	0.49	0.43	0.045

Tests of association were performed using logistic regression assuming a log-additive model adjusted for age and sex. Associations were considered significant at p < 0.05. For each significant test of association, rs number, nearest gene, originally-published associated phenotype, the current study phenotype, chromosome (CHR), coded allele (CA), odds ratio (95% confidence interval), coded allele frequency (for cases and controls), and p-value are given.

Coded allele for this study (CA).

Coded allele frequency (CAF).

Odds ratio (OR).

95% confidence intervals (CI).

Eight of the SNPs associated with RA at *p* < 0.05 were originally associated with MS (International Multiple Sclerosis Genetics Consortium, 2011; International Multiple Sclerosis Genetics Consortium et al., 2011; Patsopoulos et al., 2011; rs10866713, rs12644284, rs11962089, rs6952809) and CD (International Multiple Sclerosis Genetics Consortium et al., 2007; Franke et al., 2010; Julià et al., 2014; rs4820425, rs6556412, rs7807268, rs9286879) notably in regions outside of the MHC. The protein expressed by the *TRIM2* gene is part of the tripartite motif family that plays a role in neuroprotection, while deleterious mutations in *TRIM2* are known to cause early-onset axonal neuropathy (Ylikallio et al., 2013). SNP rs12644284 located in *TRIM2* moderately contributed to extreme MS (International Multiple Sclerosis Genetics Consortium, 2011) in a European-descent population (OR = 2.04, allele “G”), and was found to be associated with RA in this study (Table 3).

The two most significant associations in this study for CD were initially identified in a meta-analysis of European-descent populations for MS where the opposite alleles for rs1323292 (A) and *MAPK1* rs2283792 (C) contributed to MS risk (OR = 1.12 and OR = 1.10, respectively; International Multiple Sclerosis Genetics Consortium et al., 2011). Lastly, *GCH1* rs3783637(C), associated at *p* = 2 × 10^−6^ (OR = 1.10) in a GWAS conducted in a Japanese population of RA (Okada et al., 2012), was associated (*p* < 0.05) with CD in this study for the opposite allele “T.”

SNP rs2293370(T) located in an intronic region of *TIMMDC1* on chromosome 3 was associated with both MS [OR = 0.27 (CI 0.09 – 0.80); *p* = 0.017] and CD [OR = 0.25 (CI 0.07 – 0.84); *p* = 0.024] in the same directions in this study. Additionally, SNP rs3781913(A) located within the *PDE2A* gene on chromosome 11 was also found to be associated with RA [OR = 0.32 (CI 0.12 – 0.83); *p* = 0.020] and CD [OR = 0.46 (CI 0.24 – 0.91); *p* = 0.024] in the same direction.

### Joint modeling of MS, RA, and CD

By combining cases for MS, RA, and CD in a joint model, we increase power to detect an association while simultaneously testing for variants that correlate with all three phenotypes. In an ordinal regression analysis adjusted for age and sex, 22 SNPs were associated with all three phenotypes at *p* < 0.05 (Supplemental Table 5). Of the ten most significant results (Table 4), five were originally identified in CD, three in RA, and two in MS (Supplemental Table 1). Only two of these ten were located within the *HLA* region of chromosome 6 suggesting other genomic regions and pathways are contributing to autoimmunity in this study population.

**Table 4:** **The 10 most significant association results for the ordinal regression joint model adjusted by age and sex**.

**SNP**	**Gene**	**Joint Model (*p*-value)**
rs2274910	*ITLN1*	0.002
rs6457617	*HLA-DQB1*	0.004
rs1551398	*Chr 8 (intergenic)*	0.009
rs26232	*C5 or f30*	0.010
rs1323292	*RGS1*	0.015
rs6651252	*MIR1208 - MIR3686*	0.016
rs1800795	*IL6*	0.017
rs10734105	*TCERG1L*	0.017
rs7765379	*HLA-DQA2*	0.019
rs2542151	*PTPN2*	0.020

### Pathway analysis

Standard approaches for genetic association studies limit small, targeted studies due to power issues and lack of genomic coverage that in turn can hinder the identification of modest main effects and biologically relevant associations. Pathway analysis was performed with results from the joint model in order to increase power and identify pathways which may be driving generalized autoimmunity. Using PARIS, we identified two KEGG pathways that were significantly (*p* < 0.05) enriched for autoimmune disease-associated SNPs identified here. These included the NOD-like receptor signaling pathway (*p* = 0.028; Supplemental Figure 1) and the Shigellosis pathway (*p* = 0.015; Supplemental Figure 2) which contain overlapping gene elements. In healthy humans, the NOD-like receptor signaling pathway plays a role in detecting bacterial pathogens and triggering the innate immune system and localized inflammatory responses (Kanneganti et al., 2007; Shaw et al., 2008). *Shigella*, the primary cause of bacillary dysentery, effectively colonize the human intestines through a type III secretion system that pumps effector proteins into host cells that interfere with the host immune response (Ogawa et al., 2008; Schroeder and Hilbi, 2008). Strong evidence supports that infectious agents may lead to autoimmunity through a number of mechanisms including “epitope spreading” in which a long term immune response to a pathogen can lead to damage of the host cells and release of host antigens being taken up by immune cells and labeled as foreign (Vanderlugt and Miller, 2002; Ercolini and Miller, 2009).

## Discussion

In order to determine whether common autoimmune and immune-mediated diseases share an underlying genetic etiology, we performed a targeted genetic association study of common variants in MS, RA, and CD which had been implicated in previous studies. Additionally, joint modeling of the three phenotypes and pathway analysis was performed which identified overlapping KEGG pathways involved in bacterial pathogenesis. Although this study was underpowered to detect associations with moderate effect sizes below 1.80, we replicated a number of strong, well-studied associations in MS and CD.

Studies of MS have historically been confounded by the clinical heterogeneity of the disease as well as the complex interaction of genetics and environment in disease initiation, progression, and severity (Davis et al., 2013; Davis and Haines, 2015). Despite these obstacles, genetic studies identified the MHC region and the *HLA* genes as major predictors of MS over 40 years ago (1972). The HLA-DRB1^*^15 allele is a hallmark susceptibility locus for MS (Caillier et al., 2008), which is marked by rs3135388 (International Multiple Sclerosis Genetics Consortium et al., 2007). Our study replicated this association at *p* = 0.003 with an OR of 3.02 and risk allele frequency (RAF) of 25% in cases and 12% in controls, similar to another publication (OR = 2.7; RAF_cases_ = 29.8%; RAF_controls_ = 13.6%; Briggs et al., 2010).

While we replicated other associations between MS and variants in the *HLA* genes, perhaps more interesting were the inverse associations for SNPs that were identified in studies of RA. These inverse associations may represent false positive associations in the present study (a strong possibility underscoring the small sample sizes and limited power) or interesting complex pleiotropic or co-morbid relationships. SNPs rs3093024 and rs3093023 are both located in the *CCR6* gene on chromosome 6. Originally identified in independent GWAS of RA in a Chinese and Japanese population, these variants contributed to a small increase in risk of RA within these populations (OR = 1.12–1.13; Kochi et al., 2010; Jiang et al., 2014). In the BioVU European American population, these variants were associated with MS in the opposite direction [OR = 0.39–0.43]. Both MS and RA are T helper type 1 cell (Th1)—mediated AI diseases that have inconsistently been observed to co-occur in individuals at a rate varying from the general populace. In a large population study utilizing the United Kingdom General Practice Research Database, an inverse rate of comorbidity was observed between RA and MS (Somers et al., 2009). The opposite was observed in U.S.-based multiplex families with MS in which RA occurred in 2% (Barcellos et al., 2006) of cases vs. ~1% in the general U.S. population (Helmick et al., 2008). Additionally, a Taiwanese population study of MS and comorbid AI diseases also found patients diagnosed with MS were at greater risk of developing RA (OR = 4.8; Kang et al., 2010). In chart reviews from the Vanderbilt University Medical Center's EHR linked to BioVU, we noted two MS patients out of 162 (1.2%) who were diagnosed with RA. While this rate falls within the range observed in previous studies, it should be noted that the present numbers from BioVU may be biased given that medical charts were not reviewed with the intent to identify additional overlapping AI diseases.

There were a number of factors that limited the strength of this study, particularly the small number of strict cases available with genetic data. Had we accepted a more lenient definition for cases, such as including patients whose medical records contained an ICD-9-CM code for another AI disease, we could have increased the number of cases for RA (*n* = 286). By utilizing a more lenient case definition, we are able to replicate previous RA associations for rs6910071 [(Stahl et al., 2010); OR = 2.41 (CI 1.72–3.39); *p* = 3.42 × 10^−7^] and rs660895 [(Plenge et al., 2007); OR = 2.21 (CI 1.60–3.03); *p* = 9.98 × 10^−7^] at a Bonferroni significance threshold (1.5 × 10^−4^).

The strict case definitions also may have limited our ability to more fully describe the shared genetic architecture for these AI and IM outcomes. That is, the case definitions explicitly excluded patients with two or more overlapping conditions (among MS, RA, and CD). Had this exclusion not been in place, the resulting cases would be a mix of bona fide cases with two or more AI or IM conditions and those who had a billing code for one AI or IM disease but were diagnosed with another. It is well known that many of these AI and IM patients experience a diagnostic odyssey. Multiple sclerosis patients, for example, typically receive an eventual differential diagnosis where laboratory tests are ordered to rule out other conditions. More complex data mining followed by manual review of EHRs would be required to identify true cases of AI or IM for study.

Another challenge unique to EHRs compared with epidemiologic cohorts is the patient-to-patient variability related to follow-up data. For example, Davis et al. (2013) noted that the median follow-up time for MS patients in BioVU was 4.5 years, which is similar to the observed median follow-up time for BioVU patients with normal electrocardiograms (5.0 years; Denny et al., 2010) and for BioVU patients with a heart transplant (5.5 years; Oetjens et al., 2014). The range of follow-up on a per patient basis, however, can be extreme (0–20 years for MS patients; Davis et al., 2013). The extreme differences in follow-up time per patient is also reflected on the number of clinic visits available in BioVU per patient. An analysis of ~15,000 patients in BioVU revealed an average of ~82 clinic visits per patients with a wide range of one to 1456 clinic visits per patient (Crawford et al., 2015). The paucity of clinical data for a proportion of patients in any given EHR is a known caveat for use of these data in a research setting (Hersh et al., 2013).

For patients with adequate follow-up data, access to the rich, longitudinal EHR data is a strength that enables identification of patients with complex diseases that might be difficult to extract from epidemiological cohorts. Additional work, however, is required to better define clinical populations. Indeed, EHRs in the United States recently adopted the 10th revision of the International Statistical Classification of Diseases and Related Health Problems codes (ICD-10) in response to meaningful use encouraged in part by the Health Information Technology for Economic and Clinical Health (HITECH) Act as part of the American Recovery and Reinvestment Act (ARRA). Compared with ICD-9-CM, the ICD-10 billing codes expanded from 13,000 to 68,000, offering much needed granularity for both clinical care and research relevant to precision medicine.

The most significant individual SNP associations in the joint model were *HLA* variants (Table 4) and *ITLN1* (Barrett et al., 2008; Franke et al., 2010; Liu et al., 2015) and *PTPN2* (Okada et al., 2012) genes. PTPN2 is a non-receptor type tyrosine-specific phosphatase that dephosphorylates receptor and non-receptor protein tyrosine kinases in a diverse range of signaling cascades that are responsible for hematopoiesis, cell proliferation, and inflammatory response. PTPN2 also negatively regulates a number of immune pathways responsible for T-cell differentiation (Spalinger et al., 2015) and Interleukin-6 (*IL6;* Table 4) cytokine signaling (Yamamoto et al., 2002).

Consistent evidence finds that autoimmune diseases co-occur at an increased rate in family-based and population studies (Barcellos et al., 2006; Eaton et al., 2007; Somers et al., 2009; Sardu et al., 2012) suggesting that these conditions share common genetic or environmental etiologies. Here we implicate bacterial pathogenesis (e.g., NOD-like receptor signaling and Shigellosis) as a common underlying susceptibility factor for MS, CD, and RA. Individual SNPs and genes in these pathways may not be significant at a multiple-correction threshold due to small sample size and clinical heterogeneity as described previously. Yet, pathway analysis takes into consideration modest effect sizes distributed throughout genes in a pathway and increases the likelihood of identifying relevant biological components. Infectious pathogens have been hypothesized to play a major role in the development of autoimmune diseases through a number of mechanisms. A couple of these mechanisms included epitope spreading and molecular mimicry (Ercolini and Miller, 2009). Molecular mimicry results due to a pathogen carrying proteins, amino acid sequence, or antigens that closely resemble host factors which lead to T or B cells triggering an autoimmune response (Lo et al., 2000; Paludan and Bowie, 2013).

In conclusion, pathway analysis of a joint model identified overlapping KEGG pathways with implications for autoimmunity driven by bacterial pathogenesis. These pathways have already been found in individual studies of AI/IM diseases. Future studies with a larger clinical population may allow for replication and fine-tuning of these results. Longitudinal clinical or epidemiological data will be necessary to determine whether acute or chronic infections indeed lead to susceptibility or progression of comorbid AI/IM diseases.

## Author contributions

The listed authors provided substantial contributions to the conception or design of the work (NR and DC), the acquisition (DC, NR, and JM), analysis (NR and MB), or interpretation of the data (NR and DC) for the work; drafted the work (NR) or revised it critically for important intellectual context (NR, MB, JM, and DC); gave final approval of the version to be published (NR, MB, JM, and DC); and agreed to be accountable for all aspects of the work in ensuring that questions related to accuracy or integrity of any part of the work are appropriately investigated and resolved (NR, MB, JM, and DC).

### Conflict of interest statement

The authors declare that the research was conducted in the absence of any commercial or financial relationships that could be construed as a potential conflict of interest.
