# Supplementary material for: Shared Genetic Etiology of Autoimmune Diseases in Patients from a Biorepository Linked to De-identified Electronic Health Records
Source: Front Genet. 2016 Oct 20;7:185. doi: 10.3389/fgene.2016.00185 (PMC5071319; doi:10.3389/fgene.2016.00185)

Supplemental Table 1. SNPs selected from the literature and included in final association analyses of multiple sclerosis, rheumatoid arthritis, and Crohn's disease. For each SNP, the SNP ID (rs number), nearest reported gene, associated disease, variant location, associated allele, and published odds ratio (95% confidence interval) are given.

SNP ID	Reported Gene	Disease	Variation type	Allele	Published OR (95% confidence interval)
rs1000113	*IRGM*	CD	intron	T	1.30 (0.82–2.10)
rs10045431	*IL12B*	CD	intergenic	C	0.69
rs10065637	*IL6ST,IL31RA*	CD	intron	C	1.12 (1.079-1.17)
rs10181042	*PUS10*	CD	intron	T	1.14 (1.09-1.19)
rs10201872	*SP140*	MS	intron	A	1.14(1.12-1.16)
rs102275	*FADS1*	CD	intron	C	1.08 (1.04-1.12)
rs10466829	*CLECL1*	MS	missense	A	1.09 (1.08-1.11)
rs1046864	*TRAF6*	RA	UTR-3	A	0.89 (0.80-0.98)
rs10488631	*IRF5*	RA	intergenic	C	1.25 (1.14–1.37)
rs10492972	*KIF1B*	MS	intron	C	1.34 (1.23-1.48)
rs10495903	*THADA*	CD	intron	T	1.14 (1.09-1.20)
rs1062158	*NDFIP1*	MS	intron	A	1.08 (1.07-1.10)
rs10734105	*TCERG1L*	CD	intergenic	G	1.27 (1.10-1.43)
rs10761659	*ZNF365 - ALDH7A1P4*	CD	intergenic	G	1.23
rs1077667	*TNFSF14*	MS	intron	G	1.16
rs10801047	*FAM5C - RGS18*	CD	intergenic	?	1.47 (1.22-1.76)
rs10821944	*ARID5B*	RA	intron	G	1.16 (1.12-1.20)
rs10865331	*B3GNT2 - TMEM17*	CD	intergenic	A	1.10 (1.062-1.134)
rs10866713	*IL12B*	MS	intergenic	A	1.17
rs10883365	*NKX2-3*	CD	intergenic	G	1.18 (1.05-1.32)
rs10936599	*MYNN*	MS	coding	G	1.10 (1.08-1.11)
rs10947261	*HLA-DRB5,HLA-DQA1,HLA-DRB1,HLA-DRA,BTNL2*	CD	intron	T	1.36 (1.25-1.49)
rs10984447	*DBC1*	MS	intron	A	1.17 (1.09-1.25)
rs10995271	*ZNF365*	CD	intergenic	C	1.25
rs1109670	*DDEF2*	MS	intergenic	A	1.38
rs11129295	*EOMES*	MS	intergenic	A	1.10 (1.09-1.13)
rs11154801	*AHI1*	MS	intron	A	1.13

rs11167764	*NDFIP1*	CD	intergenic	C	1.06 (1.02-1.11)
rs11190140	*NKX2-3*	CD	intergenic	T	1.2
rs11195128	*SMNDC1,DUSP5,RP11-525A16.1*	CD	intergenic	T	1.42 (1.28-1.58)
rs11203203	*UBASH3A*	RA	intron	A	1.07 (1.00–1.14)
rs11209026	*IL23R*	CD	missense	G	2.66
rs11229030	*TMEM17*	CD	intergenic	C	1.15 (1.10-1.39)
rs11465804	*IL23R*	CD	intron	T	0.53
rs11581062	*SLC30A7*	MS	intron	G	1.12 (1.10-1.13)
rs11584383	*C1orf106 - KIF21B*	CD	intergenic	T	1.18
rs1160542	*LOC150577*	RA	intron	A	1.15 (1.08-1.23)
rs11747270	*IRGM*	CD	intergenic	G	1.33
rs11755724	*RREB1*	MS	intron	A	1.08 (1.06-1.09)
rs11761231	*Intergenic*	RA	intergenic	C	1.32 (female)
rs11805303	*IL23R*	CD	intron	T	1.39
rs11810217	*EVI5*	MS	intron	A	1.15 (1.13-1.16)
rs11900673	*B3GNT2*	RA	intergenic	T	1.11(1.07-1.15)
rs11962089	*POPDC3*	MS	intron	G	0.69
rs12035082	*AIMP1P2 - TNFSF18*	CD	intergenic	?	1.14 (1.02-1.27)
rs12048904	*EXTL2*	MS	intergenic	A	1.09 (1.08-1.11)
rs12212193	*BACH2*	MS	intron	G	1.09 (1.08-1.10)
rs12242110	*CREM*	CD	intergenic	G	1.15 (1.10-1.20)
rs12368653	*AGAP2, CYP27B1*	MS	intron	A	1.10 (1.09-1.12)
rs12456021	*ALPK2*	MS	intron	A	1.10 (1.08-1.12)
rs12466022	*HAAO*	MS	intergenic	C	1.10 (1.10-1.13)
rs1250550	*ZMIZ1*	CD	intron	G	1.19
rs12521868	*C5orf56*	CD	intron	T	1.23
rs12644284	*TRIM2*	MS	intron		2.04
rs12677663	*SLC43A4*	CD	intergenic	T	1.15 (1.04-1.28)
rs12720356	*TYK2,ICAM1,ICAM3*	CD	missense	G	1.12 (1.06-1.19)
rs12722489	*IL2RA*	CD	intronic	C	1.11 (1.05–1.16)
rs12831974	*TRHDE*	RA	intron	?	1.34
rs12994997	*ATG16L1, INPP5D*	CD	intron	A	1.23 (1.193-1.274)
rs13003464	*IFITM4P*	CD	intron	G	1.05 (1.00-1.40)
rs13017599	*REL*	RA	intergenic	A	1.21 (1.150-1.282)
rs13031237	*REL*	RA	intron	T	1.13 (1.07–1.18)
rs13073817	*RAD23BP1 - MIR4791*	CD	intergenic	A	1.08 (1.03-1.13)

rs13119723	*IL2,IL21*	RA	intron	A	0.87 (0.81–0.93)
rs13126505	*BANK1*	CD	intron	A	1.17 (1.10-1.248)
rs13192471	*HLA-DRB1*	RA	intergenic	G	2.1
rs13192841	*OLIG3*	MS	intergenic	A	1.10 (1.09-1.12)
rs1323292	*RGS1*	MS	intergenic	A	1.12 (1.10-1.13)
rs13333054	*IRF8*	MS	intergenic	A	1.11 (1.10-1.13)
rs1335532	*CD58*	MS	intron	A	0.78
rs13361189	*IRGM*	CD	intergenic	?	1.38 (1.15-1.66)
rs13428812	*DNMT3A*	CD	intron	G	1.06 (1.03-1.10)
rs1373692	*DAB2 - PTGER4*	CD	intergenic	?	1.46
rs1398024	*C10orf67*	CD/ sarcoidosis	intergenic	A	0.81
rs140522	*ODF3B*	MS	UTR-3	A	1.10 (1.09-1.12)
rs1456893	*Intergenic*	CD	intergenic	A	1.2
rs1456896	*IKZF1,ZPBP,FIGNL1*	CD	intergenic	T	1.14 (1.09-1.20)
rs1458175	*PDZRN4*	MS	intron	?	1.34
rs1487630	*TBC1D1*	CD	intergenic	T	1.33 (1.22-1.44)
rs151181	*IL27,SH2B1,EIF3C,LAT,CD19*	CD	intron	G	1.07 (1.03-1.12)
rs1520333	*PKIA, IL7*	MS	intergenic	G	1.10 (1.08-1.11)
rs1529316	*CSMD1*	MS	intron	?	1.35
rs1551398	*Intergenic*	CD	intergenic	A	1.08
rs1600249	*BLK*	RA	intron	?	0.77
rs1610677	*HLA-G*	RA	intergenic	A	1.32 (1.79-1.41)
rs17066096	*IL22RA2*	MS	intergenic	G	1.14
rs170934	*EOMES*	MS	intergenic	T	1.17
rs17174870	*MERTK*	MS	intron	G	1.11 (1.09-1.12)
rs17234657	*5p13.1*	CD	intergenic	G	1.77 (1.23–2.50)
rs1728918	*UCN*	CD	intergenic	A	1.12 (1.086-1.16)
rs17309827	*SLC22A23*	CD	intron	T	1.10 (1.05-1.16)
rs1736020	*NRIP1 - CYCSP42*	CD	intergenic	C	1.16 (1.11-1.21)
rs1736135	*NRIP1 - CYCSP42*	CD	intergenic	T	1.18
rs1738074	*TAGAP*	MS	UTR-5	C	1.15
rs17391694	*GIPC2 - RNFT1P2*	CD	intergenic	C	1.13 (1.077-1.194)
rs17445836	*IRF8*	MS	intergenic	G	1.25 (1.12-1.39)
rs1755289	*SH3GL2*	MS	intergenic	?	0.73
rs17582416	*PRDX2P2 - CUL2*	CD	intergenic	G	1.16
rs17594362	*KIAA0564*	MS	intergenic	A	1.11 (1.09-1.13)

rs17824933	*CD6*	MS	intron	G	1.18 (1.07-1.30)
rs1799964	*LTA,HLA-DQA2,TNF,LST1,LTB*	CD	intergenic	C	1.19 (1.13-1.25)
rs1800693	*TNFRSF1A*	MS	intron	C	1.2
rs1800896	*IL10*	Grave's disease/ CD	indel	T	1.73
rs180515	*RPS6KB1*	MS	UTR-3	G	1.09 (1.08-1.11)
rs181359	*YDJC*	CD	intron	T	1.10 (1.06-1.15)
rs1819658	*UBE2D1*	CD	intergenic	C	1.19 (1.13-1.25)
rs182549	*MCM6*	lactose intolerance	intronic	C	-
rs1841770	*ZIC1*	MS	intergenic	?	0.74
rs1847472	*BACH2*	CD	intron	G	1.07 (1.03-1.11)
rs1869839	*FOXP2*	CD	intergenic		1.20 (1.11-1.30)
rs1893217	*PTPN2*	CD	intron	G	1.25 (1.18-1.32)
rs1906493	*TRIB1*	CD	intergenic	C	1.19 (1.09-1.28)
rs1957895	*PRKCH*	RA	intron	G	1.09 (1.05-1.13)
rs1992660	*PTGER4*	CD	intergenic		1.42
rs1998598	*DENND1B*	CD	intron	G	1.04 (1.00-1.09)
rs2002842	*SALL3*	RA	intergenic	A	1.61
rs2019960	*PVT1*	MS	intergenic	G	1.12 (1.10-1.13)
rs2024092	*GPX4,HMHA1*	CD	intron	A	1.16 (1.112-1.201)
rs2058660	*IL18RAP,IL12RL2,IL18R1,IL1RL1*	CD	intron	G	1.19 (1.14-1.26)
rs2062305	*TNFSF11*	CD	intergenic	G	1.10 (1.05-1.15)
rs2062583	*ARHGEF3*	RA	intron	?	1.59 (1.30-1.92)
rs2075876	*AIRE, PFKL*	RA	intron	A	1.18 (1.11-1.24)
rs2076756	*NOD2*	CD	intron	G	1.53 (1.46-1.60)
rs2104286	*IL2RA*	MS	intronic	A/G	1.11 (0.73–1.70)
rs2119704	*GPR65*	MS	intergenic	C	1.22 (1.19-1.25)
rs212388	*TAGAP*	CD/ Celiac's disease	intergenic	C	1.10 (1.05-1.14)
rs2149085	*RNASET2, FGFR1OP, CCR6, MIR3939*	CD	intergenic	T	1.34 (1.23-1.45)
rs2150702	*MLANA*	MS	intron	G	1.16
rs2188962	*IBD5*	CD	intron	T	1.36 (1.21-1.52)
rs2230926	*OLIG3,TNFAIP3*	RA	missense	G	1.31 (1.17-1.46)
rs2233434	*NFKBIE*	RA	missense	G	1.19 (1.15-1.24)
rs2240335	*PADI4*	RA	cds-synon	A/ C	1.5
rs224136	*ZNF365 - ALDH7A1P4*	CD	intergenic	?	1.67

rs2241880	*ATG16L1*	CD	missense	G	1.45 (1.27-1.64)
rs2243123	*IL12A*	MS	intron	G	1.08 (1.06-1.10)
rs2248359	*CYP24A1*	MS	intergenic	G	1.12 (1.10-1.13)
rs2274471	*JAK2*	CD	intron	A	1.27 (1.15-1.41)
rs2274910	*ITLN1*	CD	intron	C	1.14
rs2280381	*IRF8*	RA	intergenic	T	1.12 (1.07-1.17)
rs2283792	*MAPK1*	MS	intron	C	1.10 (1.08-1.11)
rs2284553	*IFNGR2,IFNAR1,IFNAR2,IL10RB,GART,TMEM50B*	CD	intron	G	1.12 (1.086-1.162)
rs228614	*MANBA*	MS	intron	G	1.09 (1.07-1.10)
rs2293152	*STAT3*	MS	intron	C	1.22
rs2293370	*TMMDC1*	MS	intron	G	1.13 (1.11-1.15)
rs2300603	*BATF*	MS	intron	A	1.10 (1.09-1.12)
rs2300747	*CD58*	MS	intron	A	1.30 (1.14-1.47)
rs2301436	*CCR6,FGFR10P,RNASE2*	CD	intron	T	1.37 (1.22-1.53)
rs2303759	*DKKL1*	MS	intron	C	1.11(1.09-1.13)
rs231735	*CTLA4*	RA	intergenic	T	1.17 (1.11-1.23)
rs233100	*DDAH1*	MS	intergenic	G	1.08 (1.07-1.10)
rs2413583	*PDGFB - RPL3*	CD	intergenic	C	1.23 (1.17-1.29)
rs2425752	*NCOA5, CD40*	MS	intron	A	1.11 (1.10-1.13)
rs2476601	*PTPN22*	Type I Diabetes/ CD / RA	coding non-synonymous	A	1.80 (1.73-1.88)
rs2503875	*RASGEF1A - FXYD4*	MS	intergenic	A	1.66 (1.37-2.00)
rs2523393	*HLA-B*	MS	intergenic	A	1.28 (1.18-1.39)
rs2546890	*IL12B*	MS	intergenic	A	1.16
rs26232	*C5orf30*	RA	intron	C	01.14
rs2736340	*BLK*	RA	intergenic	T	1.19 (1.125-1.268)
rs2744148	*SOX8*	MS	intergenic	G	1.12 (1.10-1.14)
rs2797685	*VAMP3*	CD	intron	A	1.05 (1.01-1.10)
rs281379	*FUT2,RASIP1*	CD	intergenic	A	1.07 (1.04-1.11)
rs281380	*MAMSTR*	MS	intergenic	G	1.08 (1.07-1.09)
rs2834215	*IFNGR2*	CD	intron	?	1.22 (1.12-1.32)
rs2836754	*FLJ45139 - RPL23AP12*	CD	intergenic	C	1.15 (1.03-1.28)
rs2837960	*DSCAM - C21orf130*	RA	intergenic	G	1.05 (0.93-1.20)
rs2838519	*ICOSLG*	CD	intergenic	G	1.18 (1.13-1.23)
rs2841277	*PLD4*	RA	intergenic	T	1.15 (1.11-1.19)
rs2847297	*PTPN2*	RA	intron	G	1.10 (1.07-1.14)

rs2867461	*ANXA3*	RA	intron	A	1.13 (1.09-1.17)
rs2872507	*ORMDL3*	CD	intergenic	A	1.14 (1.09-1.19)
rs290986	*SYK*	MS	intergenic	A	1.10 (1.08-1.12)
rs2945412	*LGALS9,NOS2*	CD	intron	A	1.14 (1.110-1.175)
rs3024505	*IL10,IL19*	CD	intergenic	T	1.12 (1.07-1.17)
rs307896	*SAE1*	MS	intron	G	1.09 (1.08-1.11)
rs3091315	*CCL2,CCL7*	CD	intergenic	A	1.20 (1.14-1.26)
rs3093023	*CCR6*	RA	intron	A	1.11 (1.06–1.16)
rs3093024	*CCR6*	RA	intron	A	1.19 (1.15-1.24)
rs3094188	*PSORS1C3*	CD	intron	C	1.61 (1.33-1.94)
rs3118470	*IL2RA*	MS	intron	G	1.12 (1.10-1.13)
rs3125734	*RTKN2*	RA	missense	G	1.2 (1.13-1.27)
rs3129889	*HLA-DRB1*	MS	intergenic	G	2.97
rs3129934	*HLA-DRB1*	MS	intron	T	2.34 (1.90-2.87)
rs3135338	*HLA*	MS	intergenic	A	3.43
rs3135388	*HLA-DRA*	MS / lupus	near-gene	A	2.75 (2.46-3.07)
rs3180018	*SCAMP3,MUC1*	CD	cds-synon	A	1.13 (1.06-1.19)
rs3184504	*SH2B3*	RA	missense	C	0.92 (0.88–0.96)
rs3197999	*MST1*	CD	missense	A	1.22 (1.16-1.27)
rs354033	*ZNF767*	MS	intron	G	1.11 (1.10-1.13)
rs359457	*CPEB4*	CD	intergenic	T	1.08 (1.04-1.12)
rs3761847	*TRAF1,C5*	RA	intergenic	G	1.13 (1.08–1.18)
rs3761959	*FCRL3*	MS	intron	G	1.08 (1.06-1.09)
rs3763309	*BTNL2*	RA	intergenic	A	2.30 (2.14-2.46)
rs3780792	*VAV2*	MS	intron	G	1.60 (1.32-1.92)
rs3781913	*PDE2A*	RA	intron	T	1.12 (1.08-1.15)
rs3783637	*GCH1*	RA	intron	C	1.10 (1.06-1.14)
rs3792109	*ATG16L1*	CD	intron	A	1.34 (1.29-1.40)
rs3810936	*TNFSF15,TNFSF8*	CD	cds-synon	C	1.21 (1.15-1.27)
rs3816587	*ANAPC4*	RA	intron	C	1.09 (0.96-1.25)
rs3828309	*ATG16L1*	CD	intron	G	1.25
rs386965	*MAF*	MS	intergenic	G	1.09 (1.07-1.11)
rs3890745	*TNFRSF14*	RA	intron	T	1.12 (1.06-1.18)
rs3897478	*ADAM30*	CD	intergenic	T	1.16 (1.101-1.224)
rs397020	*C20orf46*	MS	intergenic	?	1.41
rs4075958	*RGS14*	MS	intergenic	A	1.09 (1.08-1.11)
rs4077515	*CARD9*	CD	missense	T	1.18 (1.13-1.22)
rs415890	*CCR6*	CD	intergenic	C	1.17 (1.12-1.22)
rs4263839	*TNFSF15*	CD	intron	G	1.22
rs4285028	*SLC15A2*	MS	intron	A	1.10 (1.09-1.12)
rs4308217	*CD86*	MS	intron	C	1.10 (1.08-1.11)

rs4409764	*GOT1 - NKX2-3*	CD	intergenic	T	1.22 (1.17-1.27)
rs4409785	*RGS14*	MS	intergenic	G	1.10 (1.09-1.12)
rs4410871	*MYC*	MS	intergenic	G	1.11 (1.09-1.12)
rs4613763	*PTGER4*	CD	intergenic	C	1.32
rs4648356	*MMEL1*	MS	intergenic	C	1.14 (1.12-1.16)
rs4656940	*CD244,ITLN1*	CD	intron	A	1.15 (1.09-1.21)
rs4680534	*IL12A*	MS	intergenic	C	1.12 (1.02-1.22)
rs4792814	*MAP3K14*	MS	intergenic	G	1.08 (1.06-1.09)
rs4802307	*PPP5C*	CD	intergenic	G	1.10 (1.06-1.139)
rs4809330	*RTEL1,TNFRS-F6B,SLC2A4RG*	CD	intron	G	1.12 (1.06-1.18)
rs4810485	*CD40*	RA	intron	T	1.18 (1.11-1.25)
rs4820425	*RBX1, EP300*	CD	intergenic	A	1.27 (1.17-1.38)
rs4871611	*TRIB1 - LINC00861*	CD	intergenic	A	1.17 (1.12-1.23)
rs4902642	*ZFP36L1*	CD	intergenic	G	1.07 (1.11-1.04)
rs4902647	*ZFP36L1*	MS	intergenic	G	1.11 (1.10-1.13)
rs4937362	*ETS1*	RA	intergenic	T	1.09 (1.06-1.13)
rs504963	*FUT2*	CD	UTR-3	A	-
rs516246	*DBP,SPHK2,IZUMO1,FUT2*	CD	intron	T	1.11 (1.07-1.14)
rs556560	*C5orf30*	RA	intergenic	G	0.88 (0.82-0.94)
rs5743289	*NOD2*	CD/pediatric IBD	intron	C	1.29–1.64
rs6062314	*ZBTB46*	MS	intron	A	1.16 (1.14-1.19)
rs6074022	*CD40*	MS	intergenic	C	1.15
rs630923	*CXCR5*	MS	intergenic	C	1.12 (1.10-1.14)
rs6314	*HTR2A*	RA (possibly)	coding non-synonymous	C	1.68 (1.20-2.34)
rs6448432	*chr4p15.2*	RA	intergenic	A	1.19 (1.11-1.28)
rs6457617	*HLA-DQB1region*	RA / systemic sclerosis	intergenic	T	2.36 (1.97-2.84)
rs6478106	*TNFSF15, LOC100129633, LOC645266, TNFSF8*	CD	intergenic	T	1.73 (1.60-1.86)
rs650258	*CD5, CD6*	MS	intergenic	G	1.12 (1.10-1.13)
rs651477	*EN1*	MS	intergenic	?	1.38
rs6545946	*PUS10*	CD	intergenic	C	1.16 (1.06-1.27)
rs6556412	*IL12B*	CD	intron	A	1.18 (1.13-1.24)
rs6568421	*PRDM1*	CD	intergenic	G	1.13 (1.07-1.18)
rs657075	*CSF2*	RA	intergenic	A	1.12 (1.08-1.15)
rs6596075	*SLC22A5 - C5orf56*	CD	intergenic	C	1.55 (1.00-2.39)

rs6601764	*KLF6 - AKR1E2*	CD	intergenic	C	1.16 (1.01–1.33)
rs6604026	*EVI5, RPL5*	MS	intron	G	1.15 (1.08-1.22)
rs660895	*HLA-DRB1*	RA	intergenic	?	3.62
rs6651252	*MIR1208 - MIR3686*	CD	intergenic	T	1.23 (1.17-1.30)
rs6679677	*PTPN22*	RA	intergenic	A	1.79 (1.65–1.94)
rs669607	*CMC1*	MS	intergenic	C	1.13 (1.12-1.15)
rs6716753	*SP140*	CD	intron	C	1.13 (1.089-1.18)
rs6738825	*PLCL1*	CD	intron	A	1.06 (1.02-1.11)
rs6856616	*TBC1D1,KLF3*	CD	intergenic	C	1.43 (1.31-1.57)
rs6887695	*IL12B*	CD	intergenic		1.26 (1.12-1.41)
rs6896969	*PTGER4*	MS	intergenic	C	1.10 (1.01-1.20)
rs6897932	*IL7R*	MS	missense	C	1.11 (1.09-1.13)
rs6910071	*HLA-DRB1*	RA	intron	G	2.88 (2.73–3.03)
rs694739	*PRDX5,ESRRA*	CD	intergenic	A	1.10 (1.05-1.16)
rs6952809	*CHST12*	MS	intron	A	1.08 (1.06-1.09)
rs7015630	*RIPK2*	CD	intergenic	T	1.08 (1.035-1.116)
rs703842	*METTL1, CYP27B1*	MS	intergenic	A	1.23
rs7076156	*B3GNT2*	CD	intron	G	1.19 (1.10-1.30)
rs7090512	*IL2RA*	MS	intergenic	G	1.19 (1.17-1.21)
rs713875	*MTMR3*	CD	intergenic	C	1.08 (1.04-1.13)
rs7191700	*PRM1*	MS	intergenic	C	1.15
rs7200786	*CLEC16A*	MS	intron	A	1.15 (1.13-1.16)
rs7238078	*MALT1*	MS	intron	A	1.12 (1.10-1.14)
rs7255066	*PVR*	MS	intergenic	G	1.09 (1.07-1.11)
rs736289	*SLC7A10 - CEBPA*	CD	intergenic	T	1.06 (1.02-1.11)
rs7404928	*PRKCB1*	RA	intron	T	1.08 (1.05-1.12)
rs740495	*GPX4,SBNO2*	CD	intron	G	1.16 (1.10-1.21)
rs743777	*IL2RB*	RA	intergenic	G	1.19 (1.10-1.30)
rs744166	*STAT3*	CD	intron	A	1.18
rs7517810	*TNFSF18,TNFSF4,FASLG*	CD	intergenic	T	1.22 (1.16-1.28)
rs7517847	*IL23R*	CD/ IBD	intron	?	0.62 (0.52-0.74)
rs7522462	*C1orf106, KIF21B*	MS	intron	G	1.11 (1.1-1.13)
rs7551188	*RUNX3*	CD	intron	T	1.18 (1.10-1.28)
rs7554511	*C1orf106,KIF21B*	CD	intron	C	1.14 (1.08-1.19)
rs756699	*TCF7*	MS	intergenic	A	1.12 (1.10-1.14)
rs7574865	*STAT4*	RA	intron	T	1.17 (1.10-1.25)
rs758944	*LOC100289507*	MS	intron	T	0.48 unit increase

rs7595037	*PLEK*	MS	intergenic	A	1.11(1.10-1.12)
rs762421	*ICOSLG*	CD	intergenic	G	1.13
rs7672826	*MGC45800*	MS	intergenic	?	1.36
rs7702331	*TMEM174 - FOXD1*	CD	intergenic	A	1.12 (1.07-1.17)
rs7714584	*IRGM*	CD	intergenic	G	1.37 (1.28-1.47)
rs771767	*NFKBIZ*	MS	intron	A	1.10 (1.09-1.12)
rs7746082	*PREP - PRDM1*	CD	intergenic	C	1.17
rs7765379	*HLA-DRB1*	RA	intergenic	?	2.51
rs780093	*GCKR*	CD	intron	T	1.15 (1.10-1.21)
rs7807268	*CNTNAP2 - RPL32P17*	CD	intergenic	G	1.38 (1.20–1.60)
rs7923837	*HHEX*	MS	intergenic	G	1.10 (1.08-1.11)
rs7927894	*C11orf30*	CD	intergenic	T	1.16
rs7927997	*C11orf30*	CD	intergenic	T	1.17 (1.12-1.22)
rs793108	*ZNF438*	MS	intergenic	A	1.08 (1.06-1.09)
rs8005161	*GALC,GPR65*	CD	intron	T	1.23 (1.16-1.31)
rs802734	*PTPRK*	MS	intergenic	A	1.10 (1.09-1.12)
rs8049603	*HS3ST2 - USP31*	MS	intergenic	T	1.19
rs805297	*APOM*	RA	intergenic	A	1.56 (1.36-1.80)
rs806321	*DLEU1*	MS	intergenic	A	1.08 (1.07-1.10)
rs8112449	*CDC37, TYK2*	MS	intergenic	G	1.08 (1.07-1.10)
rs864745	*CREB5,JAZF1*	CD	intron	T	1.09 (1.052-1.123)
rs874628	*MPV17L2*	MS	intron	A	1.11(1.09-1.12)
rs881375	*TRAF1, C5*	RA	intergenic	?	-
rs882300	*CXCR4*	MS	intergenic	C	1.19 (1.09-1.30)
rs908821	*SLC25A36*	MS	intergenic	?	1.37
rs9258260	*HLA-F, MOG, HLA-G, GABBR1, HLA-H, UBD, HLA-A*	CD	intergenic	T	1.45 (1.21-1.68)
rs9267911	*PBX2, NOTCH4*	CD	intergenic	T	1.50 (1.29-1.76)
rs9268853	*HLA-DRA*	RA	intergenic	C	2.40(2.20-2.60)
rs9271366	*DQA1*	MS	intergenic	G	2.62 (2.09-3.28)
rs9272219	*HLA-DQA1*	RA	intergenic	-	-
rs9282641	*CD86*	MS	intron	G	1.21(1.18-1.24)
rs9286879	*AIMP1P2 - TNFSF18*	CD	intergenic	G	1.19
rs9292777	*DAB2-PTGER4*	CD	intergenic	T	1.37 (1.28-1.48)
rs9296015	*HLA locus*	RA	intergenic	?	-
rs931555	*IL7R*	MS	intron	?	1.25 (1.15-1.36)
rs9321490	*MYB*	MS	intergenic	G	1.10 (1.08-1.11)

rs9348876	*AIF1*	CD	intergenic	T	1.41 (1.22-1.63)
rs9469220	*HLA-DQB1 - HLA-DQA2*	CD	intergenic	A	1.14 (0.98–1.32)
rs951005	*CCL21*	RA	intergenic	A	1.19
rs9523762	*GPC5*	MS	intron	?	1.36
rs9657904	*CBLB*	MS	intron	T	1.40 (1.27-1.57)
rs9821630	*PLCL2*	MS	intron	G	1.08 (1.07-1.10)
rs9858542	*BSN, MST1*	CD	cds-synon	A	1.09 (0.96-1.24)
rs9891119	*STAT3*	CD	intron	A	1.37 (1.27-1.48)

"?" SNPs in which the allele was not listed in the GWAS Catalog (http://www.ebi.ac.uk/gwas/)

Supplemental Table 2. The 50 most significant results for Crohn's disease. Single SNP tests of association were performed using logistic regression assuming a log additive model adjusted by age and sex. For each test of association, the SNP (rs number), chromosome location, coded allele, odds ratio, 95% confidence interval (CI upper and lower bounds), and p-value are given.

SNP	CHR	Coded Allele	OR	CI-lower	CI-upper	P-value
rs1323292	1	C	2.8	1.486	5.276	0.001451
rs2283792	22	T	2.586	1.333	5.018	0.004973
rs7714584	5	G	2.663	1.227	5.779	0.01326
rs3780792	9	G	0.6177	0.4141	0.9215	0.01823
rs2293370	3	T	0.244	0.07324	0.8127	0.02157
rs13119723	4	G	0.2039	0.04721	0.8803	0.03311
rs3781913	11	A	0.4841	0.2456	0.9543	0.03616
rs7238078	18	G	0.6169	0.3921	0.9705	0.03668
rs3783637	14	T	1.586	1.017	2.472	0.04177
rs10181042	2	T	0.5084	0.2641	0.9786	0.04291
rs2002842	18	A	1.448	1.007	2.08	0.04547
rs6596075	5	G	0.565	0.3175	1.006	0.05225
rs1736020	21	A	0.7289	0.527	1.008	0.05607
rs13333054	16	T	1.894	0.9807	3.659	0.05719
rs233100	1	T	1.389	0.9847	1.959	0.06122
rs5743289	16	T	1.936	0.9689	3.87	0.0614
rs10188217	2	T	1.392	0.9814	1.974	0.06365
rs6908425	6	T	0.6326	0.3894	1.028	0.06442
rs2274910	1	T	0.5126	0.2521	1.043	0.06504
rs7574865	2	T	1.603	0.9562	2.688	0.07345
rs630923	11	A	0.2657	0.06181	1.142	0.07491
rs10866713	5	A	0.5646	0.2999	1.063	0.07657
rs10045431	5	A	0.7008	0.472	1.04	0.07784
rs3761959	1	A	0.5686	0.3021	1.07	0.08014
rs2024092	19	A	0.4871	0.2131	1.113	0.08809
rs12644284	4	G	0.7083	0.4741	1.058	0.09225
rs2076756	16	G	1.368	0.9438	1.982	0.09807
rs6651252	8	C	0.3693	0.113	1.207	0.09923
rs281380	19	C	0.548	0.2657	1.13	0.1035
rs6457617	6	C	1.331	0.9378	1.89	0.1094
rs3828309	2	C	1.803	0.8716	3.732	0.112
rs10466829	12	G	1.642	0.8882	3.036	0.1137

rs9286879	1	G	0.5657	0.2733	1.171	0.1249
rs10995271	10	C	1.697	0.8392	3.43	0.1411
rs1551398	8	C	0.7613	0.5278	1.098	0.1445
rs3816587	4	C	1.385	0.893	2.148	0.1458
rs881375	9	T	1.312	0.9088	1.894	0.1472
rs2523393	6	C	1.379	0.8886	2.14	0.1517
rs4792814	17	T	1.584	0.8292	3.026	0.1637
rs2872507	17	A	1.272	0.9048	1.788	0.1664
rs3093024	6	A	0.7822	0.5523	1.108	0.1665
rs3180018	1	A	0.5885	0.2761	1.254	0.1698
rs3764147	13	G	1.314	0.8876	1.947	0.1724
rs6601764	10	C	1.272	0.8988	1.801	0.1744
rs2019960	8	C	1.306	0.886	1.925	0.1774
rs3890745	1	G	1.267	0.8981	1.787	0.1776
rs2062583	3	G	0.5854	0.2676	1.281	0.1801
rs7927894	11	T	1.586	0.8001	3.144	0.1864
rs744166	17	C	0.7873	0.5512	1.124	0.1885
rs6856616	4	C	2.121	0.686	6.558	0.1917

Supplemental Table 3. The 50 most significant results for multiple sclerosis. Single SNP tests of association were performed using logistic regression assuming a log additive model adjusted by age and sex. For each test of association, the SNP (rs number), chromosome location, coded allele, odds ratio, 95% confidence interval (CI upper and lower bounds), and p-value are given.

SNP	CHR	Coded Allele	OR	CI-lower	CI-upper	P-value
rs2301436	6	A	0.3818	0.2289	0.6367	0.000225
rs3093024	6	A	0.3936	0.2337	0.6628	0.000454
rs11581062	1	G	0.3695	0.1969	0.6935	0.001939
rs3093023	6	A	0.3321	0.1611	0.6845	0.002814
rs9271366	6	G	2.164	1.294	3.621	0.003269
rs3135388	6	T	3.041	1.407	6.572	0.004673
rs3129889	6	G	3.041	1.407	6.572	0.004673
rs2274910	1	T	0.3619	0.1695	0.7726	0.008617
rs3129934	6	T	1.94	1.159	3.247	0.01172
rs2836754	21	T	1.784	1.137	2.8	0.01185
rs13126505	4	A	3.099	1.263	7.6	0.01348
rs2243123	3	C	2.136	1.16	3.936	0.01486
rs2293370	3	T	0.2644	0.08846	0.7901	0.01723
rs2523393	6	T	2.777	2.5020	3.0801	0.01834
rs2546890	5	A	1.7500	1.3200	2.3200	0.02268
rs1610677	6	G	2.223	1.118	4.422	0.0228
rs12368653	12	A	1.988	1.1	3.594	0.02283
rs4409785	11	C	1.818	1.069	3.09	0.02725
rs6457617	6	T	0.6005	0.3757	0.9599	0.0331
rs13031237	2	T	1.923	1.05	3.521	0.03416
rs874628	19	C	1.629	1.036	2.562	0.03455
rs7255066	19	C	1.643	1.036	2.608	0.035
rs13017599	2	A	1.919	1.043	3.53	0.03606
rs12048904	1	T	0.4824	0.2426	0.9593	0.03766
rs281380	19	C	0.4553	0.2163	0.9587	0.03835
rs1520333	8	C	0.4224	0.1854	0.962	0.04016
rs2076756	16	G	0.5246	0.2825	0.9743	0.04112
rs26232	5	T	1.98	1.026	3.819	0.04169
rs9268853	6	C	0.4041	0.1678	0.9729	0.04326

rs6448432	4	A	0.4821	0.2365	0.9828	0.04467
rs7090512	10	C	1.648	1.012	2.683	0.0447
rs9286879	1	G	0.4607	0.2143	0.9904	0.04717
rs2300603	14	C	2.235	1.006	4.969	0.04841
rs10995271	10	C	0.1301	0.01613	1.049	0.05547
rs1000113	5	T	2.837	0.9547	8.434	0.06059
rs1077667	19	A	1.655	0.9688	2.827	0.06517
rs7765379	6	G	0.3821	0.1369	1.067	0.06624
rs228614	4	G	1.5384	0.4019	1.037	0.07041
rs17594362	13	T	1.932	0.946	3.946	0.07065
rs17391694	1	T	1.833	0.9398	3.574	0.07542
rs2274471	9	C	1.725	0.9438	3.152	0.07643
rs660895	6	G	0.5564	0.2897	1.068	0.07822
rs17582416	10	G	1.668	0.9351	2.976	0.08315
rs13003464	2	G	1.488	0.9479	2.336	0.08409
rs12456021	18	A	0.4348	0.1685	1.122	0.08504
rs1800693	12	G	1.493	0.941	2.368	0.08882
rs7522462	1	A	0.532	0.2563	1.104	0.09023
rs908821	3	C	1.562	0.9307	2.623	0.09136
rs2476601	1	A	0.4073	0.1422	1.167	0.09438
rs9891119	17	C	0.5646	0.2857	1.116	0.1

Supplemental Table 4. The 50 most significant results for rheumatoid arthritis. Single SNP tests of association were performed using logistic regression assuming a log additive model adjusted by age and sex. For each test of association, the SNP (rs number), chromosome location, coded allele, odds ratio (OR), 95% confidence interval (CI upper and lower bounds), and p-value are given.

SNP	CHR	Coded Allele	OR	CI-lower	CI-upper	P
rs3781913	11	A	0.3137	0.1181	0.8332	0.02001
rs7765379	6	G	2.405	1.127	5.129	0.02323
rs10866713	5	A	4.811	1.199	19.31	0.02672
rs4820425	22	A	2.105	1.053	4.21	0.03522
rs6556412	5	A	2.237	1.044	4.792	0.03834
rs7807268	7	G	0.1161	0.0143	0.9374	0.04332
rs12644284	4	G	0.3484	0.1249	0.9718	0.04394
rs6952809	7	T	0.3741	0.1417	0.9879	0.0472
rs11962089	6	G	3.103	1.012	9.513	0.04761
rs9286879	1	G	1.921	0.9784	3.773	0.0579
rs1800896	1	G	1.862	0.9744	3.558	0.05991
rs2274910	1	T	0.4267	0.1724	1.056	0.06537
rs4937362	11	T	1.794	0.9600	3.353	0.06693
rs9321490	6	C	0.2633	0.06136	1.13	0.07252
rs7927894	11	T	3.017	0.8800	10.34	0.07898
rs660895	6	G	1.795	0.9094	3.545	0.09176
rs2301436	6	A	1.801	0.9004	3.604	0.09622
rs386965	16	G	0.4127	0.1441	1.182	0.09917
rs7404928	16	C	1.768	0.8975	3.483	0.09947
rs1458175	12	T	1.771	0.8953	3.502	0.1006
rs3764147	13	G	1.764	0.8943	3.481	0.1014
rs3197999	3	T	1.828	0.8789	3.803	0.1064
rs12466022	2	A	1.699	0.8783	3.287	0.1154
rs11190140	10	T	0.5829	0.2878	1.18	0.1338
rs6908425	6	T	1.726	0.8372	3.558	0.1393
rs881375	9	T	0.5632	0.2609	1.216	0.1436
rs1992660	5	G	0.4296	0.1356	1.362	0.1511

rs4680534	3	C	0.6024	0.2997	1.211	0.1547
rs3135388	6	T	2.671	0.6842	10.43	0.1574
rs3129889	6	G	2.671	0.6842	10.43	0.1574
rs2546890	5	G	1.596	0.8342	3.052	0.1579
rs10758669	9	C	1.571	0.8382	2.946	0.1587
rs11900673	2	T	0.2426	0.03303	1.782	0.1639
rs802734	6	C	2.379	0.7003	8.079	0.1649
rs12521868	5	T	0.6187	0.3100	1.235	0.1734
rs10761659	10	A	2.487	0.6664	9.278	0.1751
rs2188962	5	T	0.6197	0.3103	1.238	0.1752
rs4409764	10	T	0.3851	0.09667	1.534	0.1761
rs805297	6	T	0.2398	0.02996	1.919	0.1784
rs2248359	20	T	0.6066	0.2913	1.263	0.1815
rs6910071	6	G	1.646	0.7822	3.463	0.1893
rs224136	10	T	0.379	0.08781	1.636	0.1934
rs7255066	19	C	1.56	0.7969	3.055	0.1944
rs504963	19	C	0.6437	0.3294	1.258	0.1977
rs9858542	3	A	0.2508	0.02947	2.134	0.2055
rs4809330	20	A	1.654	0.7546	3.624	0.209
rs11761231	7	T	2.248	0.6347	7.96	0.2094
rs17594362	13	T	1.721	0.7319	4.045	0.2134
rs1109670	2	A	1.532	0.778	3.017	0.2172
rs3828309	2	C	2.155	0.6286	7.386	0.222

Supplemental Table 5. The 50 most significant results from the MultiPhen Joint Model. Ordinal regression models were adjusted by age and sex. For each result, the SNP (rs number), p-values for individual tests of association by phenotype (Crohn's disease or CD; multiple sclerosis or MS, and rheumatoid arthritis or RA), and p-values for the joint model are given.

	Individual phenotype tests of associations (p-values)			
SNP	CD	MS	RA	Joint Model
rs2274910	0.178	0.269	0.243	0.002
rs6457617	0.836	0.669	0.208	0.004
rs1551398	0.421	0.537	0.699	0.009
rs26232	0.236	0.213	0.211	0.010
rs1323292	0.577	0.561	0.253	0.015
rs6651252	0.284	0.473	0.234	0.016
rs1800795	0.003	0.003	0.003	0.017
rs10734105	0.012	0.014	0.738	0.017
rs7765379	0.907	0.631	0.262	0.019
rs2542151	0.036	0.020	0.811	0.020
rs1738074	0.292	0.595	0.264	0.022
rs9348876	0.470	0.020	0.029	0.022
rs4149584	0.229	0.287	0.226	0.025
rs2523393	0.502	0.827	0.603	0.028
rs6856616	0.021	0.709	0.044	0.030
rs233100	0.930	0.559	0.243	0.034
rs2002842	0.784	0.779	0.222	0.039
rs6908425	0.610	0.751	0.277	0.042
rs10181042	0.367	0.611	0.494	0.042
rs2076756	0.483	0.557	0.971	0.046
rs1520333	0.319	0.966	0.470	0.048
rs12048904	0.802	0.565	0.357	0.048
rs10865331	0.243	0.568	0.805	0.054
rs1000113	0.264	0.463	0.409	0.055
rs9286879	0.909	0.538	0.349	0.056
rs2240335	0.268	0.420	0.296	0.058
rs12131057	0.030	0.935	0.043	0.060
rs9271366	0.443	0.895	0.293	0.063
rs281380	0.858	0.326	0.868	0.064

rs2945412	0.227	0.306	0.472	0.064
rs771767	0.795	0.480	0.415	0.067
rs6952809	0.322	0.472	0.483	0.068
rs6596075	0.300	0.448	0.604	0.068
rs11581062	0.315	0.515	0.278	0.069
rs102275	0.265	0.644	0.550	0.071
rs212388	0.245	0.318	0.326	0.074
rs13031237	0.411	0.851	0.462	0.077
rs3780792	0.346	0.381	0.244	0.081
rs2062305	0.378	0.592	0.305	0.084
rs13017599	0.424	0.821	0.486	0.088
rs11900673	0.347	0.315	0.927	0.090
rs793108	0.389	0.299	0.835	0.092
rs12644284	0.319	0.264	0.731	0.096
rs881375	0.409	0.499	0.403	0.099
rs7574865	0.721	0.375	0.401	0.101
rs1600249	0.694	0.416	0.451	0.102
rs13192471	0.595	0.750	0.328	0.107
rs5743289	0.513	0.533	0.648	0.110
rs1957895	0.427	0.296	0.316	0.111

Supplemental Figure 1: NOD-like Receptor Signaling Pathway. Pathway (map04621) from the Kyoto Encyclopedia of Genes and Genomes (KEGG) at http://www.genome.jp/kegg/.

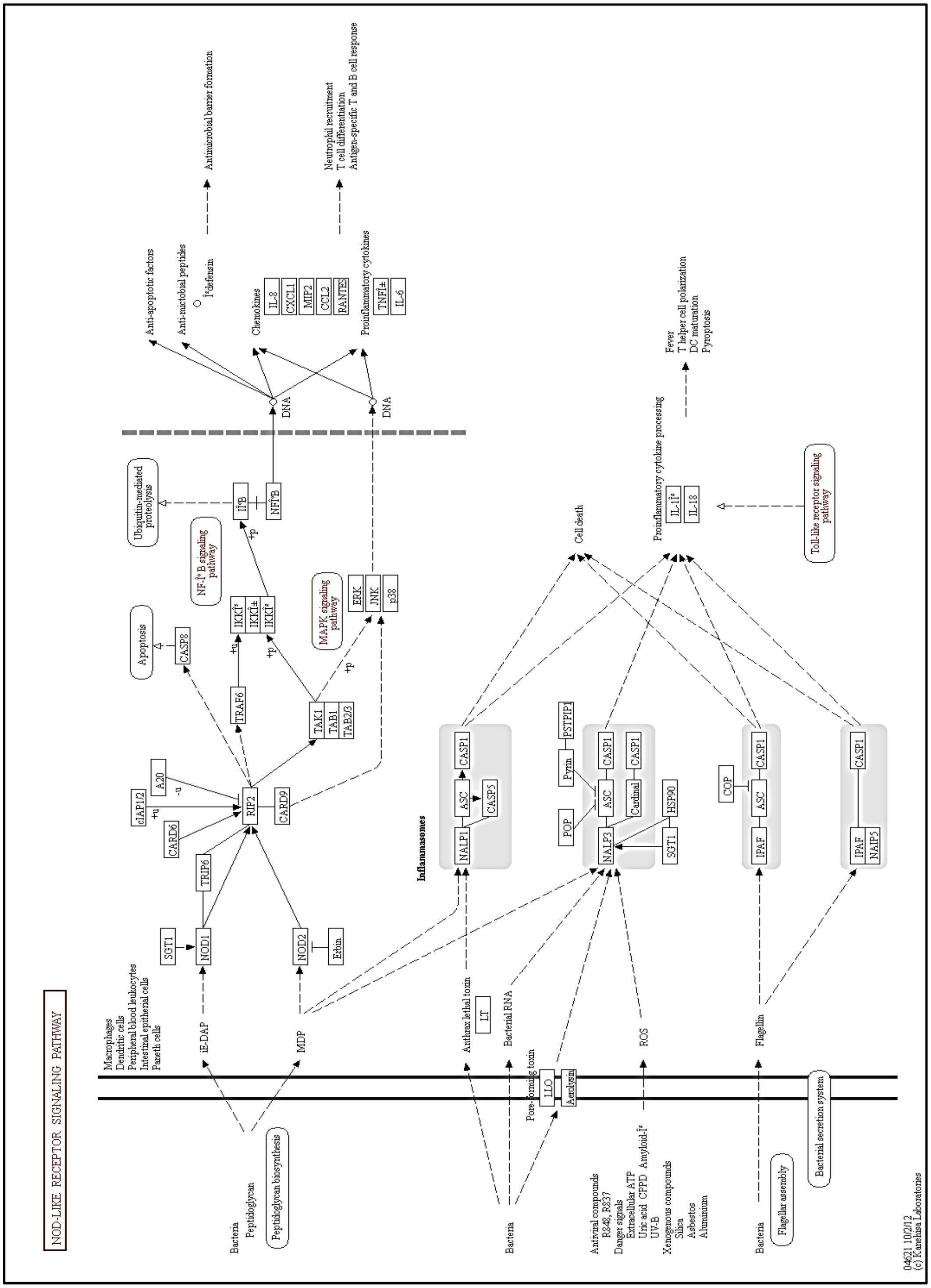

Supplemental Figure 2: Shigellosis Pathway. Pathway (map05131) from the Kyoto Encyclopedia of Genes and Genomes (KEGG) at http://www.genome.jp/kegg/.

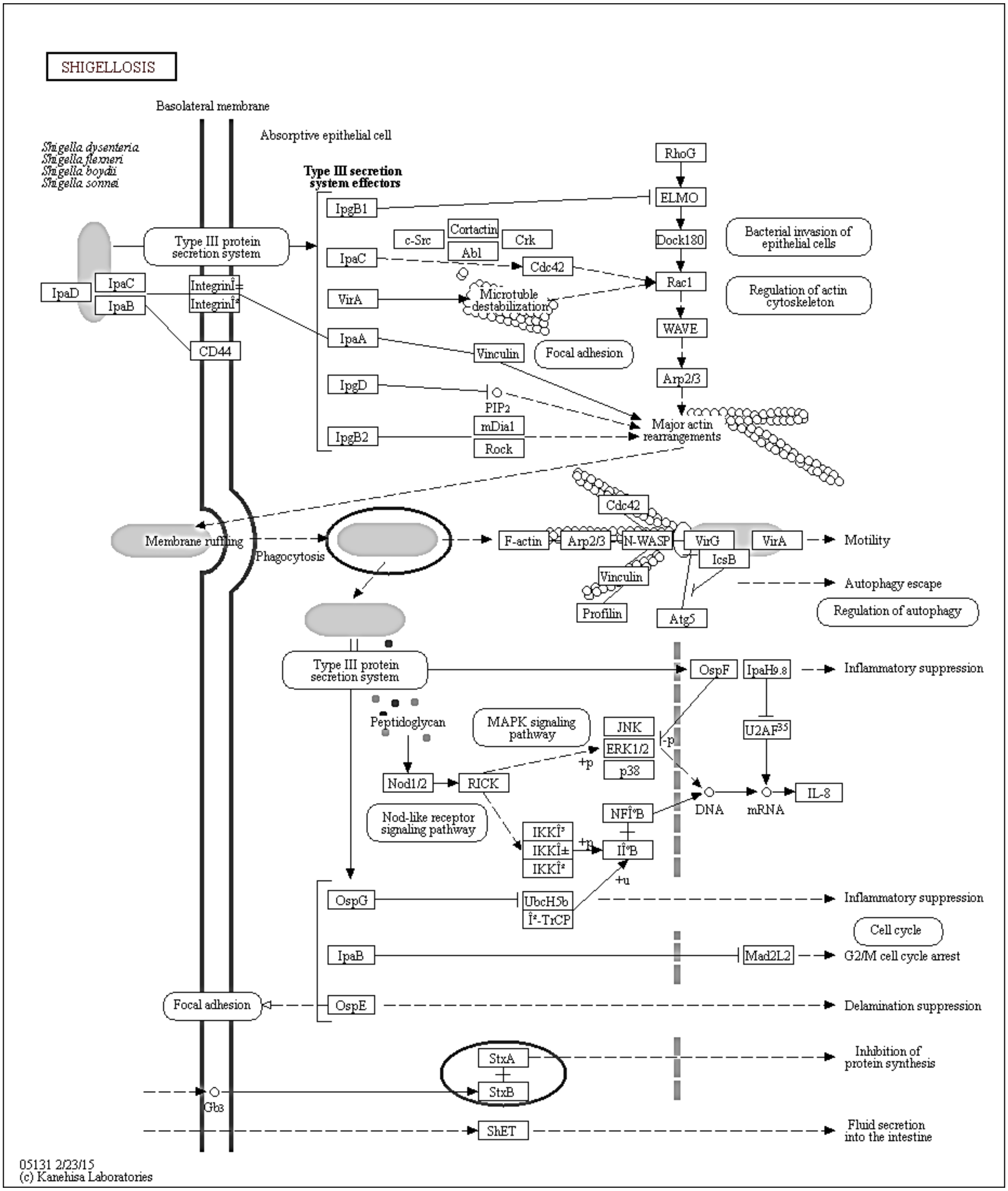

Supplement: Supplementary file 1 [file Table1.PDF]
